# Virtual Viewing Time: The Relationship between Presence and Sexual Interest in Androphilic and Gynephilic Men

**DOI:** 10.1371/journal.pone.0127156

**Published:** 2015-05-18

**Authors:** Peter Fromberger, Sabrina Meyer, Christina Kempf, Kirsten Jordan, Jürgen L. Müller

**Affiliations:** Ludwig-Meyer-Institute for Forensic Psychiatry and Psychotherapy, Faculty of Medicine, Georg-August-University, Göttingen, Lower Saxony, Germany; University of Vienna, AUSTRIA

## Abstract

Virtual Reality (VR) has successfully been used in the research of human behavior for more than twenty years. The main advantage of VR is its capability to induce a high sense of presence. This results in emotions and behavior which are very close to those shown in real situations. In the context of sex research, only a few studies have used high-immersive VR so far. The ones that did can be found mostly in the field of forensic psychology. Nevertheless, the relationship between presence and sexual interest still remains unclear. The present study is the first to examine the advantages of high-immersive VR in comparison to a conventional standard desktop system regarding their capability to measure sexual interest. 25 gynephilic and 20 androphilic healthy men underwent three experimental conditions, which differed in their ability to induce a sense of presence. In each condition, participants were asked to rate ten male and ten female virtual human characters regarding their sexual attractiveness. Without their knowledge, the subjects’ viewing time was assessed throughout the rating. Subjects were then asked to rate the sense of presence they had experienced as well as their perceived realism of the characters. Results suggested that stereoscopic viewing can significantly enhance the subjective sexual attractiveness of sexually relevant characters. Furthermore, in all three conditions participants looked significantly longer at sexually relevant virtual characters than at sexually non-relevant ones. The high immersion condition provided the best discriminant validity. From a statistical point of view, however, the sense of presence had no significant influence on the discriminant validity of the viewing time task. The study showed that high-immersive virtual environments enhance realism ratings as well as ratings of sexual attractiveness of three-dimensional human stimuli in comparison to standard desktop systems. Results also show that viewing time seems to be influenced neither by sexual attractiveness nor by realism of stimuli. This indicates how important task specific mechanisms of the viewing time effect are.

## Introduction

“The ultimate display would, of course, be a room within which the computer can control the existence of matter. […] such a display could literally be the Wonderland into which Alice walked.” (p. 508) [1] Even tough this vision of Ivan Sutherland has not yet become reality, Virtual Realities (VR) are already successfully used in a wide variety of contexts, such as assessment and treatment of psychiatric disorders, medical staff training and empirical research of complex human behavior [2, 3]. The most important advantages of VR are the high ecological validity of Virtual Environments (VE), the extensive controllability of stimuli in the VE and of course, the possibility of creating a sensation of actually being in the VE instead of the real physical environment [4]. As a result, VR can elicit emotional responses which are similar to those in real life situations [5]. All these features of VR are what makes it possible to improve both the internal and external validity of experimental designs, especially in empirical studies of human behavior [6].

From a behavioral point of view, VR can be defined as an advanced form of human-computer interface, that allows the user to interact with and become immersed in a computer-generated environment in a naturalistic fashion [7–9]. This computer-generated environment is called virtual environment (VE). This definition picks up two main features of VR: immersion and presence. Immersion “refers to the degree of physical stimulation impinging on the sensory systems and the sensitivity of the system to motor inputs.” (p. 475) [10] Thus, immersion is only an objective description of aspects of the technological system and can be increased, for example, by widening the field of view or by increasing the resolution of screens. Presence, on the other hand, is a psychological phenomenon and can be defined as the feeling of being in one place or environment even when one is physically situated in another [11]. In other words, presence is the feeling of being in the virtual environment [2, 7, 12]. An exhaustive overview about the concept of presence can be found in the explication statement of the International Society for Presence Research [13]. The concept of presence has been considered as central in virtual reality research, because it is assumed that higher presence results in the same emotions and reactions within a VE which would be expected in a similar real-world situation [14]. Some studies suggest that sense of presence experienced in a VE could even be higher than in the real world [15].

In current research, at least two components of presence are distinguished: *Spatial presence* and *Involvement*[11, 16]. Two factor analytic studies based on 246 and 296 participants, [17] identified an additional third component, *Realness*. Spatial presence describes what is commonly included in the definition of presence whereas Involvement focuses on attentional processes, such as focus on the VE and suppression of the real environment. Realness concerns a comparison between the VE and the real world [11, 17, 18].

### Factors influencing the sense of presence

A multitude of factors are assumed to influence the perceived intensity of presence in VE’s. The include, for example, technical aspects like stereoscopic or monoscopic viewing, resolution of screen, field of view, tracking of head and body movements, as well as user characteristics like susceptibility for motion sickness or psychiatric disorders [19]. One aspect, which is mentioned as one of the most important factors, is interactivity or the ability to interact with the VE [20, 21]. Interactivity can be defined as the degree to which users can modify and change the form or content of the virtual environment [20]. Assuming that presence results from the cognitive representation of possible actions that can be performed in the virtual world, one can argue that interactivity could influence (spatial) presence [22]. A study by Regenbrecht et al. provide empirical evidence for this assumption. [22]. The authors had 65 healthy subjects experience a virtual environment with an HMD in one of two experimental conditions. The participants were either able to move freely through the VE or they were shown a prerecorded sequence from a first-person perspective. Results suggested that the possibility to move within the VE significantly increased spatial presence and realism of the VE. Welch et al. [23] examined the impact of realism of the textures and interactivity on the sense of presence in a driving simulation. Interactivity was manipulated by either letting subjects virtually drive a car or making them a passive passenger. They found that both factors significantly increased presence, whereas pictorial realism was more influential than interactivity. Nevertheless, it has to be mentioned that the importance of realism with regards to sense of presence was not supported in other studies, which could lead to the conclusion that realism is not as important for presence as other factors like interactivity [3]. In a study by Hendrix et al. [24], interactivity was operationalized by head tracking. Twelve male participants viewed a virtual room and either could or could not change the viewpoint in real-time. Further experimental manipulations concerned stereoscopy (stereoscopic or monoscopic viewing) and the field of view. Results showed that the possibility to interact with the virtual environment and stereoscopic viewing significantly enhanced the feeling of being part of the VE. Sense of realism was only enhanced by head tracking, not by stereoscopic viewing. Furthermore, wider fields of view resulted in significantly higher presence and realism ratings. The authors concluded that the possibility to interact with the environment, stereoscopy and the FOV are important factors for a high sense of presence in virtual environments, whereas stereoscopy does not seem to be the most important factor.

A study by Freeman et al. [25], despite its small sample size, hints at another important aspect which could contribute to sense of presence: Stereoscopy. Stereoscopy provides an illusion of depth in three-dimensional contents by presenting a different image to each eye [26]. In their experiment, the authors showed three film sequences to a sample of twelve healthy subjects and manipulated the viewing condition (stereoscopic viewing versus monoscopic viewing). Their results showed a main effect for this manipulation regarding presence ratings, demonstrating that subjective feelings of presence were enhanced by stereoscopic stimulus presentation. Other studies too suggested that stereoscopic viewing can significantly enhance the subjective sense of presence [26]. For example, Ijsselstein et al. [27] presented a video to 24 healthy participants which was presented on a screen in an either monoscopic or stereoscopic fashion. The subjective presence was higher when stimuli were viewed stereoscopically than when stimuli were viewed monoscopically. This result emphasize once more the positive impact of stereoscopy on sense of presence.

### The relationship between presence and induced emotions

There seems to be no doubt in literature that subjective sense of presence is related to emotional reactions elicited by a VE [26, 28]. For example, Riva et al. [29] compared emotional reactions evoked by a neutral, an anxious and a relaxing virtual environment based on a sample of 61 healthy participants. Results showed that the different environments induced different emotions in the expected direction: the anxious condition induced highest negative emotions whereas the relaxing condition induced highest positive emotions in comparison to the neutral condition. Furthermore, the level of presence was significantly higher in the anxious and in the relaxing condition than in the neutral condition. Banos et al. [30] randomly assigned 80 healthy subjects to five VEs, which differed regarding their induced emotional state (sad, happy, anxious, relaxed and neutral). The authors did not use a high-immersive VR system. Instead, they presented the virtual environment on a large screen without stereoscopic viewing. The subjective induced emotional state and the sense of presence was significantly higher in the emotional VEs than in the neutral VE. Juan et al. [31] compared the relationship between presence and anxiety in two VR systems which differed regarding their amount of immersion. 25 healthy participants were immersed in a virtual environment which was made to induce anxiety. Results suggested that the more immersive the VR system, the higher the sense of presence and anxiety. Kim et al. [32], too, showed a positive relationship between presence and emotion. The authors used emotionally evocative virtual environments in order to induce stress. 35 healthy participants were confronted with a stressful and a non-stressful VE in three different VR systems, which differed regarding their immersiveness. In contrast to the studies mentioned above, emotional responses were assessed with an objective psychophysiological measure (skin conductance). Results revealed that the highest subjective arousal was reported in the most immersive system, whilst the least immersive system caused the lowest subjective arousal. Skin conductance responses and sense of presence differed between the VR systems in the same manner. The higher the technological immersiveness, the higher the skin conductance response and the higher the subjective sense of presence. The authors concluded that a higher sense of presence comes along with a higher emotional arousal.

Nevertheless, the causality of the relationship between presence and induced emotions still remains unclear [11]. For example, Frijda [33] assumed that the degree of emotions corresponds to realism of perceived events assuming a causal relationship between the sense of realism and emotional reactions. The higher the realism, the higher the emotional reaction. Recent studies suggest that emotion is a necessary prerequisite for experiencing presence [29, 34]. For example, Riva et al. [29], who’s study was mentioned above, additionally calculated a Multilevel Linear Regression analysis on their data, which showed that level of presence was a significant predictor of emotional state. The authors concluded that affective responses in virtual environments may generally be lower without a high feeling of presence [29].

### Virtual environments in the context of sex research

Given the above-mentioned advantages and the ability of high-immersive VE with regards to inducing emotions, surprisingly few studies have used VR in the field of sexology so far. Sexual experiences can be defined as an emotional experience, including the awareness of autonomic arousal, expectation of reward, and motivated desire [35]. From this point of view, high-*immersive* VR could be a unique tool in sex research as well as in psychotherapeutic treatment of sexual disorders. Recently, this potential of VR systems was recognized in the context of forensic research, regarding (risk) assessment and therapy of pedophiles and child abusers [4, 36, 37].

Renaud et al. [38], for example, explored the usability of virtual characters presented in a high-immersive VE for the assessment of pedophilic deviant interests. 30 male child abusers and 29 male non-deviant subjects were put into a VR with animated virtual characters (male and female adult, male and female child, neutral character with no texture) for 90 seconds while penile plethysmography (PPG) and eye movements were recorded. Results showed, that child abusers showed significantly higher penile responses when facing child characters, whereas the control group showed significantly higher penile responses when facing adult virtual characters. An analysis of eye movements showed that child molesters looked significantly longer at sexual features of all virtual characters than control subjects did. The authors concluded that *immersive* virtual reality in combination with psychophysiological measures can be a powerful tool for the assessment of deviant sexual interests, especially due to the high ecological validity of virtual characters. Also in prior studies, Renaud and colleagues found distinct motor and oculomotor patterns which were linked to deviant and non-deviant sexual interests in subjects who were immersed in VR’s with sexual stimuli [39–41].

To our knowledge, there are only two studies which used high-immersive VE to study sexual experiences in healthy men. Renaud et al. [42] measured eye movements and penile responses of 20 healthy gynephilic male participants while they were immersed with virtual sexual stimuli. In order to explore the perceptual-motor correlates of sexual self-regulation abilities, participants were divided into two groups according to their capacity to focus attention while being immersed (high and low focus). Each subject took part in three experimental conditions. In one condition, the subject was presented with a virtual woman and was instructed to not inhibit the sexual response. In the second condition, the subject was also presented with a virtual woman but was this time instructed to inhibit the sexual response. In the third and neutral condition, the subject was presented with a neutral virtual character without a colored skin or genitals. Results suggested that the erectile responses were significantly higher in the “no inhibition” condition than in the “neutral” or “inhibition” condition. Thus, the experimental intervention was validated. Furthermore, eye movements showed a significant difference between the conditions: The inhibition condition showed significantly less eye movement transitions in the genital zone than were found in the neutral or the no inhibition condition. Regarding the total dwell time in the genital zone of the virtual character, there was no significant difference between the experimental conditions. The authors inferred that eye movements reflect the modulation of attention which is involved in the inhibition of sexual arousal. Regarding group differences, the high-focus group showed significantly stronger erectile responses than the low-focus group, even though this effect was only found in the “inhibition” condition. The authors summarized that high-*immersive* VEs are a methodological improvement over static and moving pictures, since they make it possible to study the role of social (sexual) interactions in an ecologically valid and well-controlled manner.

In a further study with a similar experimental design, Trottier et al. [43] immersed 20 healthy subjects with five different virtual human characters (male and female adult, male and female child, neutral character with no texture). As in the studies mentioned above, eye movements and penile responses were measured. There were three different instructions: (a) free visual exploration of a preferred sexual stimulus without erectile inhibition; (b) viewing of a preferred sexual stimulus with erectile inhibition and (c) free visual exploration of a non-preferred sexual stimulus. To inhibit the erectile response, subjects were instructed to memorize a disgusting image. Results showed that attempts to control erectile responses generate specific eye-movement variations, characterized by a general deceleration of the exploration process and less exploration of the erogenous zone. The authors conclude that recording eye movements can provide important information on the presence of competing covert processes responsible for erectile inhibition.

These studies show the many possibilities and the basic usability of high-immersive VE in sex research. Up to now, only one study addressed this question in the context of child abusers. Renaud et al. [37] compared high-immersive virtual characters with auditive stimuli and showed that high-immersive visual stimuli surpassed auditive stimuli regarding their effectiveness in inducing sexual arousal based on PPG-data. 22 child abusers and 42 non-deviant adult males took part in the study. While both stimulus modalities generated significantly different genital arousal profiles for child abusers and non-deviant males, deviance differentials calculated from the VR modality provided significantly higher classification accuracy. The authors conclude that in comparison to audio stimuli, the VR system makes it possible to improve not only accuracy of group classification but also discriminant validity. This provides empirical support for the use of this new method for PPG assessment. Yet, the comparison of different modalities can not sufficiently answer the question, if high immersive VR can induce stronger sexual experiences than conventional non-immersive desktop systems. Knowledge about this relationship would be important because it could provide information regarding the intensity of immersion necessary for virtual sex research environments. To our knowledge, however, there exists no study which explores the assumed predominance of high-immersive VR systems over conventional desktop systems in the induction of sexual interest directly.

### Viewing Time as an objective measure of sexual interest

Besides self reports and interviews, sexual research uses a wide range of methods to objectively assess sexual interest or sexual arousal. These include PPG [44], eye movements [45] and other cognitive oriented approaches [46]. Viewing Time (VT), for example, is a common method, especially used by clinicians, to assess deviant sexual interests. Several commercial screening methods exist for this purpose already, e. g. the Abel Assessment for Sexual Interest [47] or Affinity [48]. The VT method goes back to Rosenzweig [49], who first reported a correlation between the time people spend on sexually attractive pictures and their sexual orientation. In the past 70 years, a multitude of empirical data replicated these effects and showed that gynephilic men take significantly more time to look at female pictures than at male pictures, whereas androphilic men look longer at male pictures than female pictures [50–55]. In most studies, the measurement of the time people look at pictures is combined with a rating task. The participant has to rate sexually relevant and sexually non relevant pictures in terms of sexual attractiveness. The time between stimulus onset until the end of the sexual attractiveness rating is called viewing time [52]. The so called viewing time effect which is typically found when viewing time is assessed, shows that participants take a significantly longer time to look at sexually attractive pictures than at sexually non attractive ones. This effect is able to indicate not only the sexual orientation, but also the sexual age preferences of the participant (e.g. the sexual interest in children) [47, 48, 56]. Since the measurement of the viewing time is not obvious for the participant, it is assumed that the tendency of participants to answer in a socially desirable manner cannot influence the viewing time results. However, the mechanisms underlying the viewing time effect are, up to now, not well examined. Two different possible explanations are discussed: stimulus-driven and/or task-specific mechanisms [51, 52]. Stimulus driven concepts assume that the sexual salience of a stimulus mainly influences the viewing time. The higher the sexual saliency the longer the viewing time. Task-specific mechanisms suggest that the task of rating sexual attractiveness involves cognitive demands, e.g. decisions about the physical features or the sex of the target person which are assumed to have the most impact on viewing time. Recent studies suggest that stimulus-specific as well as task-specific processes contribute additively to the viewing time effect. Task-specific mechanisms seem to have a higher impact than stimulus-driven aspects. Nevertheless, up to now, it remains unclear, which kind of task-specific or stimulus-driven processes take place in detail [52].

### Aim of the Study and Hypotheses

This study aims to explore the relationship between presence and sexual interest for three-dimensional virtual characters. In oder to do so, we used VT to assess sexual interest and transferred the VT method into a three-dimensional VE. One of the most important factors of virtual reality is the ability to induce a feeling of being in the virtual environment. As described in detail above, some studies could lead one to the conclusion that higher sense of presence should increase the induced emotion. If sexual interest is defined as an emotional response, higher presence should result in higher sexual interest and attraction. Stereoscopy and interactivity are able to enhance the feeling of actually being in virtual environments, which should result in higher (subjective and objective) sexual interest in sexually relevant virtual characters. By comparing the viewing time in VEs which differ regarding their ability to induce presence, it will be possible to explore the influence of presence on sexual interest.

Taken the above-mentioned studies into account, we hypothesize that (1) stereoscopy as well as interactivity result in a higher subjective feeling of being there. We assume further, that (2) a higher feeling of being there results in significantly higher subjective sexual attractiveness ratings of sexually relevant virtual characters. Regarding the viewing time, we hypothesize that (3) subjects look significantly longer at sexually relevant virtual characters than at sexually non-relevant characters. This should hold true for viewing time with and without stereoscopy and interactivity. Finally, we assume that (4) a higher sense of presence results in a more pronounced viewing time effect, which should become apparent in a higher discriminant validity (5).

## Methods

### Participants

A total of 25 gynephilic (*M* = 26.92 years, *SD* = 7.79) and 20 androphilic healthy (*M* = 30.85 years, *SD* = 10.20) males participated in study. The groups did not differ regarding their age (*t*(34.87) = 1.42, *p* = .164) or educational level (*U*(25, 20) = 235.00, *p* = .430). All participants were recruited by a notice posted on the campus of the University of Göttingen or by contacting gay groups in Göttingen via Email. Sexual orientation was assessed by the Kinsey scale asking for physical contacts [57], accepting only ratings from 0 to 1 (exclusively or predominantly gynephilic) or 5 to 6 (exclusively or predominantly androphilic). All participants were without history of neurological or psychiatric illness according to the DSM-IV [58]. All participants had normal visual acuity or to normal corrected visual acuity. Furthermore, all participants provided written informed consent before participating in the study. The study was approved by the ethics committee of the medical faculty at Georg-August-University of Göttingen.

### Material

#### Stimuli

**Fig 1:**
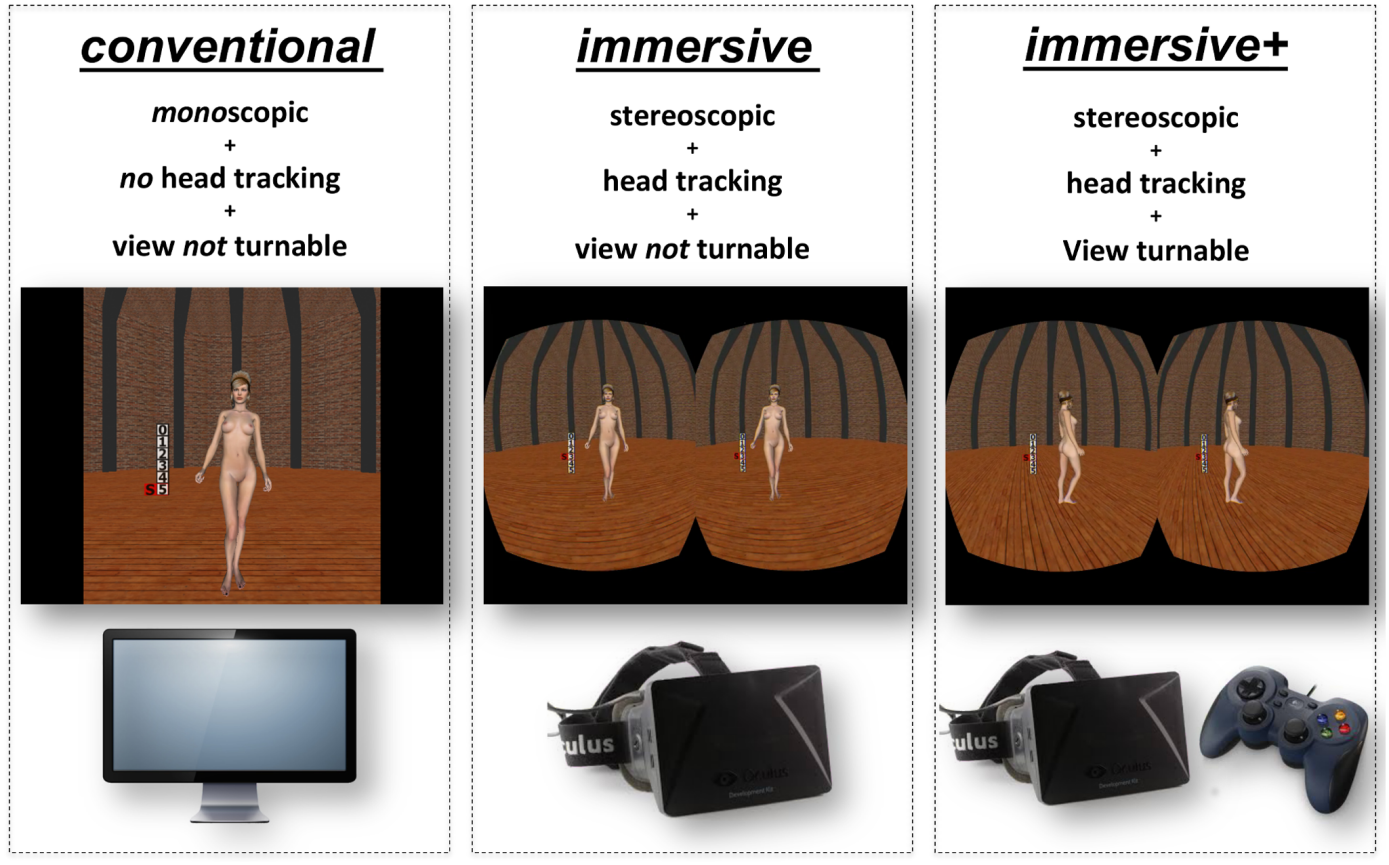
Exemplary illustration of two trials. In each trial, the participant had the task to rate a virtual human character first regarding sexual attractiveness and afterwards regarding realism. Throughout the attractiveness rating, the time from stimulus onset until the rating was measured without the knowledge of the subject. All in all, there were 20 trials, ten trials showing male characters and ten trials presenting female characters. The order of the trials was fully randomized.

Stimuli were computer-generated realistic, fully rigged three-dimensional (3-D) virtual characters of naked human beings (see Fig 1 for an exemplary male and female virtual character). All virtual characters were modeled and designed at the University of Göttingen. The work-flow for modeling and designing the characters started with modeling of the characters with the 3D modeling software Poser (Version 9; Smith MicroSoftware, Inc., Watsonville, CA) and DAZ Studio Pro (Version 4.6; DAZ Productions, Inc., Salt Lake City, UT). All characters were taken from the free example female and male models, which came with the DAZ Studio Pro. After designing different body shapes, the models were exported to 3ds Max (Version 2014; Autodesk, Inc.) and optimized regarding geometry, rigging and posing. Finally, textures were designed with the 2D graphical software tool GIMP (Version 2.8; www.gimp.org). A total of 20 computer-generated characters was designed, ten male and 10 female characters. The characters differed regarding their body shape, hair shape and texture. Within each gender group of the characters, the pose was identical. All characters were modeled with a friendly face directly looking in the direction of the viewer. The characters were not animated.

#### Apparatus

Two different hardware setups were used in the study. The virtual reality system comprised a head-mounted display (HMD; Oculus Rift Development Kit 1, Oculus VR, Inc., Irvine, CA) with a resolution of 1280 × 800 pixel (vertical FOV: 100°). The HMD provides stereoscopic viewing by presenting a separate picture to each eye of the participant, resulting in an effective resolution of 640 × 800 pixel per eye. Head-movements of the participants were assessed by an integrated 3-Degree-of-Freedom (DOF) movement sensor. Non-stereoscopic presentation of the stimuli was achieved using a standard 19 inch TFT-monitor (1907FP, Dell Inc., Round Rock, TX) with an adjusted resolution of 640 × 800 pixel. Thus, the resolutions of the two presentation systems were identical. Interaction with the virtual environment and the subjective ratings were done with the help of a gamepad (Logitech F301: Logitech Inc, Newark, CA). All experiments were scripted with WorldViz Vizard Toolkit (Version 4; WorldViz LLC., Santa Barbara, CA), a python-based virtual reality software.

### Measures

#### Simulator Sickness Questionnaire (SSQ)

The SSQ [59] is a self-report questionnaire to measure typical symptoms of simulator sickness. Its symptoms are similar to to those of motion-induced sickness, but originate from elements of the visual display and visuo-vestibular interaction [60, 61]. The questionnaire consists of 16 items based on a four-point Likert scale ranging from 0 (the symptom is not existent) to 3 (very severe symptom). The SSQ consisted of three distinct symptom clusters (Oculomotor, Disorientation, and Nausea) and a total score. The total score measures the overall severity of simulator sickness.

#### Igroup Presence Questionnaire (IPQ)

The IPQ [17] is a self-report questionnaire to measure the sense of presence in virtual reality environments. It contains 14 items rated on a seven-point Likert scale ranging from 0 to 6. The IPQ contains three sub-scales that measure different components of presence (see introduction section for more information): (1) the Spatial Presence sub-scale is related to the sense of physically being in the VE, (2) the Involvement sub-scale is meant to evaluate the attention devoted to the VE, and (3) the Realness sub-scale evaluates the sense of reality attributed to the VE. Additionally, the IPQ contains one general item which assesses the general “sense of being there”, and has high loadings on all three factors, with an especially strong loading on Spatial Presence. The IPQ provides a high reliability (Cronbach’s *α* = .87).

#### Subjective realism, sexual attractiveness, and objective sexual interest

Subjective realism of the virtual characters and sexual attraction elicited by the virtual characters were assessed with a six-point Likert scale ranging from 0 (not at all realistic / not at all sexually attractive) to 5 (very realistic / very sexually attractive) while looking at the virtual characters. The objective sexual interest was assessed using the viewing time method. Traditionally, for this method, subjects are asked to rate sexually relevant and sexually non relevant two-dimensional pictures of humans regarding sexual attractiveness while the time they take to look at the pictures is assessed. The time between stimulus onset and the end of the sexual attractiveness rating is defined as viewing time [48, 50–52, 56].

### Procedure

**Fig 2:**
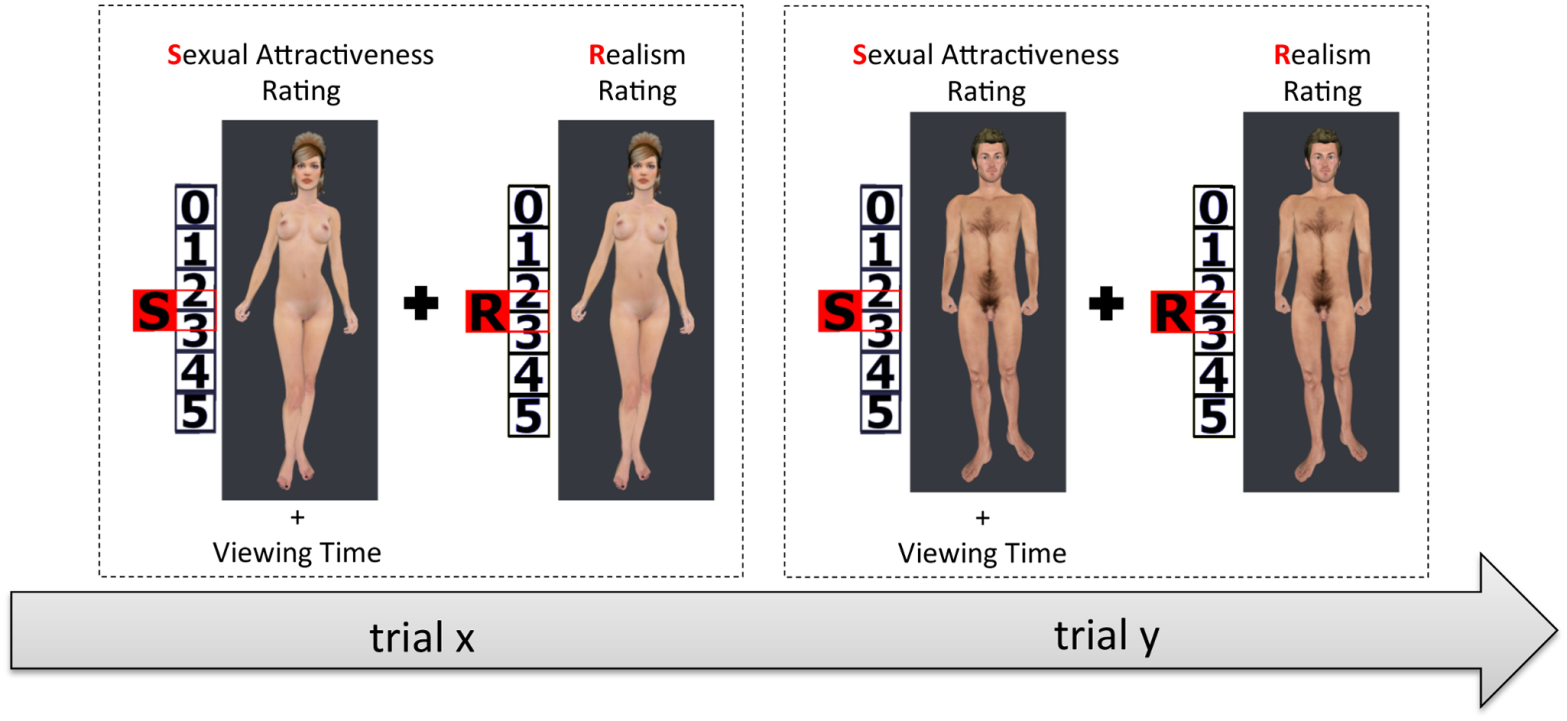
Schematic overview of the three experimental conditions. In the *conventional* condition virtual characters were presented on a standard desktop PC enabling only monoscopic viewing, no head tracking and no interactivity. In the *immersive* condition a Head Mounted Display (HMD) was used, enabling stereoscopic viewing and head tracking. The *immersive+* condition used the same technical equipment as the *immersive* condition, but additionally allowed the participant to turn around the virtual human characters. Note that the procedure was exactly the same in all three experimental conditions.

The experiment consisted of three main experimental conditions. All participants took part in all conditions. The order of the experimental conditions was balanced across the participants using the Latin square method in order to avoid sequence effects. In all three experimental conditions, the procedure was exactly the same, but they differed regarding their degree of immersion. The degree of immersion was manipulated by providing stereoscopic viewing and interactivity. In the *conventional* condition, the virtual characters were presented on a standard TFT-monitor without the possibility to interact with the environment and with only monoscopic viewing. The participant had the task to rate the virtual characters regarding sexual attractiveness while the response time was measured unobtrusively. Thus, the *conventional* condition was an exact replication of the classical viewing time method [47, 48, 52, 56]. In contrast, in the *immersive* condition, the virtual characters were presented by an HMD, enabling stereoscopic viewing and interactivity by head tracking. At last, in the *immersive+* condition, the participant saw the virtual characters in a stereoscopic manner (HMD), head movements were tracked and applied to the VE in real time, and the subject had the opportunity to turn around the virtual character. Fig 2 illustrates the differences between the three experimental conditions.

When arriving at the lab, the participants were briefed in the purpose of the study. After signing the consent form, a short questionnaire was applied in order to asses neurological or psychiatric disorders. The participants were then asked to fill in the Kinsey Scale [57] and the pre-test of the SSQ. Afterwards, the participants were seated alone in a quiet room facing the monitor or wearing the HMD. The investigator was able to monitor the progress of the experiment by a self-written program (scripted in Python 2.7.6; www.python.org) in a separate room. All three experimental conditions were conducted consecutively with a little rest between them.

In all three experimental conditions, the procedure was exactly the same: Each experimental condition was divided into three phases. In the instruction phase, the participant read the instruction via monitor or HMD. The participant was told that he has the task to rate the virtual characters regarding their sexual attractiveness and regarding their realism on a 6-point Likert scale (0 = not sexually attractive at all / not realistic at all, 5 = very sexually attractive / very realistic). The instruction emphasized that the subjective feeling of the participant was of interest, rather than how he thinks others would feel about the character. In the *immersive+* condition he was additionally told, that he has the opportunity to turn around the virtual character before making his choice. After the instruction phase, the participant walked through six test trials (three clothed male and three clothed female characters) in order to get comfortable with the task and with the controlling of the experiment. The six test characters were not presented in the main experimental phase. The test trials followed the same rationale as the main trials afterwards. Each experimental phase consisted of 20 trials, ten trials with male characters and ten trials with female characters (see Fig 1 for the experimental design). The order of the trials was fully randomized. Each trial started with an automatic opening of a curtain (duration: 2 seconds). Only now, the participant saw a wooden stage and the virtual character in the center of the stage. On the right side of the character, a visual rating scale was presented with a red marker between the second and third value of the rating scale in order to avoid biasing the participant. The letter “S” on the marker indicated that the sexual attractiveness should be rated (see Fig 2 for the virtual environment). Without the knowledge of the participant, the time from stimulus onset until the end of the sexual attractiveness rating was assessed in each trial (Viewing Time). After choosing the corresponding value on the rating scale using the cross of the gamepad and pressing the B-Button of the gamepad to confirm the choice, the first rating scale was replaced by a new rating scale. This rating scale was identical to the first one except the letter “R” was shown instead of the letter “S”, indicating the start of the realism rating. After confirming the choice of the realism rating, the curtain closed (duration: 2 seconds) and the next trial started. After the experimental phase, the subject was asked to fill in the post-test of the SSQ in order to assess possible symptoms of the simulator sickness. Additionally, he was asked to fill in the IPQ in order to asses the subjective sense of presence.

### Data Analysis

Data analysis was performed with the statistic software R (Version 3.1.2) [62]. All dependent variables were analyzed with linear mixed models using the R package “lme4” [63]. Degrees of freedom and p-values were estimated with the Kenward-Roger approximation (R package “afex”) [64]. Significant main effects or interactions were further analyzed with post-hoc pairwise comparisons based on the Least squares means (R package “lsmeans”) [65]. The Tukey method was used to adjust for multiple comparisons. The significance level was set to *α* = .05. The rationale of the linear mixed models analyses followed the guideline of [66] and [67]. The authors showed that models with maximum random effects (but still identifiable) fit best for our experimental design.

A linear mixed model with Subject Group (androphilic vs. gynephilic), Experimental Condition (*conventional* vs. *immersive* vs. *immersive+*) and Time point (pre vs. post) as fixed factors and random intercepts, that were different for each individual subject at each Time point and at each Experimental condition, fitted the SSQ data best. For the IPQ, a linear mixed model with Subject Group and Experimental Condition as fixed factors and a random intercept, that was different for each individual subject, fitted the data best. Due to misunderstanding of the general item, the SSQ for one gynephilic subject could not be analyzed. A linear mixed model with Subject Group, Experimental Condition and Gender of virtual character (male vs. female) as fixed factors and the main effects and interaction of the within-subject factors as random slopes, that were different for each subject, and a random intercept, that was different for each virtual character, best fitted the viewing time and rating data (realism and sexual attractiveness rating). Since viewing time data is known for having outliers due to distraction of the subject, outliers within the viewing time data were identified following the approach of [68] and deleted. This approach considers the sample size and calculates outliers for each subject in each condition individually. All in all, 93 outliers were identified. In order to analyze to what extend subjects interacted with the virtual environment in the immersive+ condition, we assessed the sum of degrees subjects turned around the character. A linear mixed model with Subject Group and Gender of virtual characters as fixed factors and main effect of the within-subject factor as random slope, that was different for each subject, and a random intercept, that was different for each virtual character, best fitted the turn around data.

Receivers operating characteristic (ROC) analyses were performed in order to determine how well the three experimental conditions differentiated between gynephilic and androphilic participants. Classifier performance was measured by the area under the curve (AUC). To do so, a Sexual Orientation Index (SOI) was calculated and defined following the approach of [69] as the difference between female and male characters. The higher the SOI, the higher the viewing time for female characters in comparison to the viewing time for male characters. For the SOI, a linear mixed model with Subject Group and Experimental Condition as fixed factors and a random intercept, that was different for each individual subject, was performed. ROC analysis was applied if the SOI differed significantly between gynephilic and androphilic subjects in the respective experimental condition. The cut-off criterion in the ROC analysis was determined following the approach of [70]. The optimal thresholds (optimal cut-off points) were defined as the threshold that maximizes the distance to the identity line.

## Results

### Simulator Sickness

Table 1 shows the means and SDs for the SSQ *Total score* as a function of Group, Condition, and Time point. In order to explore if the experimental conditions enhanced simulator sickness symptoms, the SSQ was applied before and after each experimental condition. The linear mixed model for the *Total score* of the SSQ revealed neither a significant main effect nor a significant interaction (see Table 2). Thus, wearing a HMD did not resulted in enhanced simulator sickness symptoms.

**Table 1:** Means and SDs for the SSQ Total score as a function of subject Group, experimental Condition, and Time point.

Condition	gynephilic				androphilic			
	prae		post		prae		post	
	*M*	*SD*	*M*	*SD*	*M*	*SD*	*M*	*SD*
*conventional*	2.12	2.94	1.83	2.60	2.33	3.68	2.39	3.85
*immersive*	2.08	3.05	3.04	3.75	2.83	4.48	3.56	4.91
*immersive+*	2.38	2.67	2.62	2.84	2.39	3.85	3.06	4.60

The Simulator Sickness Questionnaire (SSQ) [59]*Total score* is defined as the sum of all 16 SSQ items. Each item is based on a four-point Likert scale ranging from 0 (the symptom is not existent) to 3 (very severe symptom).

**Table 2:** Results of the linear mixed model with Subject Group, Experimental Condition and Time point as fixed factors and random intercepts, that were different for each individual subject at each Time point and at each Experimental condition, for the SSQ Total score.

Fixed effect	*ndf/ddf* ^*d*^	*F* ^*d*^	*p* ^*d*^
Group^*a*^	1/66.24	.25	.62
Timepoint^*b*^	1/119.71	.46	.50
Condition^*c*^	2/133.99	2.34	.10
Group × Time point	1/119.71	.28	.60
Group × Condition	2/133.99	.01	.99
Time point × Condition	2/80.00	2.18	.12
Group × Time point × Condition	2/80.00	.31	.74

^*a*^gynephilic subjects vs. androphilic subjects.

^*b*^pre-measure vs. post-measure.

^*c*^conventional vs. immersive vs. immersive+.

^*d*^Kenward-Roger approximation.

### Sense of presence

Table 3 shows means and SDs of the IPQ sub-scales as a function of subject Group and Condition. Table 4 shows the results of the linear mixed models. The linear mixed model for the *General item (“sense of being there”)* only revealed a significant main effect for Condition, *F*(2,84) = 40.12, *p* <.001. Post-hoc pairwise comparisons showed in line with hypothesis 1, that the *conventional* condition resulted in a significantly lower feeling of being there than the *immersive* condition, *t*(92.19) = -8.48, *p* <.001, *d* = 1.74, or the *immersive+* condition, *t*(92.19) = -10.49, *d* = 2.19, *p* <.001. Against hypothesis 1, the *immersive+* and the *immersive* condition did not differ significantly, *t*(92.19) = -2.01, *p* = .12, *d* = .42.

**Table 3:** Means and SDs for the Igroup Presence Questionnaire (IPQ) [17] as a function of subject Group and Condition.

Subscale	Condition	gynephilic		androphilic	
		*M*	*SD*	*M*	*SD*
General item^*a*^	*conventional*	.52	1.36	1.11	1.82
	*immersive*	3.24	1.83	3.53	2.06
	*immersive+*	3.88	1.72	4.11	1.94
Spatial presence^*b*^	*conventional*	3.33	.47	3.38	.41
	*immersive*	3.23	.55	2.98	.66
	*immersive+*	3.06	.67	3.07	.67
Involvement^*c*^	*conventional*	2.17	.59	2.67	.74
	*immersive*	3.41	.71	3.35	.89
	*immersive+*	3.64	.71	3.59	.68
Realness^*d*^	*conventional*	2.68	.61	2.67	.69
	*immersive*	3.02	1.21	3.35	.92
	*immersive+*	3.11	1.32	3.59	.50

The IPQ comprises 14 items rated on a seven-point Likert Scale ranging from 0 to 6.

^*a*^The general item assesses the general “sense of being there”, and has high loadings on all three factors, with an especially strong loading on Spatial Presence.

^*b*^The Spatial Presence sub-scale is related to the sense of being physically inside the VE.

^*c*^The Involvement sub-scale is directed to evaluate the attention devoted to the VE.

^*d*^The Realness sub-scale evaluates the sense of reality attributed to the VE.

**Table 4:** Results of the linear mixed models with Subject Group and Experimental Condition as fixed factors and a random intercept, that was different for each individual subject, for all sub-scales of the IPQ.

Sub-scale	Fixed effect	*ndf/ddf* ^*c*^	*F* ^*c*^	*p* ^*c*^
General presence	Group^*a*^	1/98.36	1.17	.28
	Condition^*b*^	2/84	40.12	***
	Group × Condition	2/84	.20	.82
Spatial Presence	Group^*a*^	1/126.81	.01	.940
	Condition^*b*^	2/86	3.63	.030
	Group × Condition	2/86	1.11	.330
Involvement	Group^*a*^	1/81.37	5.52	.020
	Condition^*b*^	2/86	65.17	***
	Group × Condition	2/86	2.58	.080
Experienced Realism	Group^*a*^	1/84.19	.23	.630
	Condition^*b*^	2/86	43.64	***
	Group × Condition	2/86	.37	.690

^*a*^gynephilic subjects vs. androphilic subjects.

^*b*^conventional vs. immersive vs. immersive+.

^*c*^Kenward-Roger approximation.

*** *p* <.001.

The linear mixed model for the sub-scale *Spatial presence*, too, only revealed a significant main effect for condition, *F*(2,86) = 3.63, *p* = .03. Post-hoc pairwise comparisons showed that the *conventional* condition resulted in a significantly lower spatial presence than the *immersive* condition, *t*(86) = -3.59, *p* = .002, *d* = .77, or the *immersive+* condition, *t*(86) = -3.73, *p* = .001, *d* = .80. There was no significant difference between the *immersive+* and the *immersive* condition, *t*(86) = -.14, *p* = .989, *d* = .03.

The linear mixed model for the sub-scale *Involvement* revealed a significant main effect for condition, *F*(2,86) = 65.17, *p* <.001, and a significant main effect for Subject group, *F*(1,81.37) = 5.52, *p* = .020. Gynephilic subjects experienced higher involvement than androphilic subjects. Post-hoc pairwise comparisons showed that the *conventional* condition resulted in a significantly lower involvement than the *immersive* condition, *t*(86) = -9.85, *p* <.001, *d* = 2.12, or the *immersive+* condition, *t*(86) = -12.51, *p* <.001, *d* = 2.70. In line with hypothesis 1, the *immersive+* results in a significant higher involvement than the *immersive* condition, *t*(86) = -2.66, *p* = .025, *d* = .57.

The linear mixed model for the sub-scale *Experienced Realism* revealed only a significant main effect for condition, *F*(2,86) = 43.64, *p* <.001. Post-hoc pairwise comparisons showed that the *conventional* condition resulted in a significantly lower involvement than the *immersive* condition, *t*(94.19) = -9.02, *p* <.001, *d* = 1.86, or the *immersive+* condition, *t*(94.19) = -10.91, *p* <.001, *d* = 2.25. But there was no significant difference between the *immersive+* and the *immersive* condition, *t*(94.19) = -1.89, *p* = .148, *d* = .39.

### Realism of virtual characters

Means and SDs for the subjective ratings of the realism of the virtual characters are provided in Table 5. The linear mixed model revealed a significant main effect for Subject group, *F*(1,43) = 6.10, *p* = .020, and a significant main effect for Condition, *F*(2,41.90) = 20.89, *p* <.001. All other main effects or interactions were not significant (see Table 6). Androphilic subjects rated virtual characters altogether as significantly more realistic than gynephilic subjects. Post-hoc pairwise comparisons further showed, that virtual characters were perceived as significantly more realistic in the *immersive+* condition than in the *conventional*, *t*(47.10) = -7.19, *p* <.001, *d* = 2.10, or in the *immersive* condition, *t*(47.05) = -4.08, *p* <.001, *d* = 1.19. Also, virtual characters were rated as significantly more realistic in the *immersive* condition than in the *conventional* condition, *t*(47.13) = -4.82, *p* <.001, *d* = 1.14.

**Table 5:** Means and SDs for the realism rating, sexual attractiveness rating, and Viewing time as a function of subject Group, Condition, and Gender of virtual character.

Measure	Condition	gynephilic				androphilic			
		female		male		female		male	
		*M*	*SD*	*M*	*SD*	*M*	*SD*	*M*	*SD*
Realism^*a*^	conventional	1.97	1.10	1.87	1.35	2.23	1.18	2.56	1.11
	immersive	2.56	1.09	2.65	1.17	2.47	1.21	2.96	0.95
	immersive+	2.92	1.07	2.92	1.10	2.66	1.02	3.23	0.87
Attractiveness^*b*^	conventional	2.14	1.31	.29	.99	.70	1.19	2.60	1.44
	immersive	2.69	1.31	.32	1.06	.82	1.19	2.94	1.35
	immersive+	2.88	1.27	.29	1.00	.81	1.22	3.15	1.31
Viewing Time^*c*^	conventional	4082.39	3017.83	2288.56	1733.01	2635.05	2578.41	4658.49	4559.03
	immersive	4478.64	3335.43	2531.39	1948.80	3118.32	2604.14	5432.89	4015.05
	immersive+	10744.15	6809.89	5293.08	4350.35	5174.57	4468.56	10482.60	10411.03

^*a*^Absolute range, 0 (not at all realistic) to 5 (very realistic).

^*b*^Absolute range, 0 (not at all sexually attractive) to 5 (very sexually attractive).

^*c*^in milliseconds (ms).

**Table 6:** Results of the linear mixed models with Subject Group, Experimental Condition and Gender of virtual character as fixed factors and the main effects and interaction of the within-subject factors as random slopes, that were different for each subject, and a random intercept, that was different for each virtual character, for Realism rating, Sexual attractiveness rating, and Viewing Time.

		Realism			Attractiveness			Viewing Time	
Fixed effect	*ddf/ndf* ^*d*^	*F* ^*d*^	*p* ^*d*^	*ddf/ndf* ^*d*^	*F* ^*d*^	*p* ^*d*^	*ddf/ndf* ^*d*^	*F* ^*d*^	*p* ^*d*^
Group^*a*^	1/43	6.10	.020	1/43	60.89	***	1/43	9.44	.004
Gender^*b*^	1/57.41	.18	.670	1/59.01	34.72	***	1/45.96	15.27	***
Condition^*c*^	2/41.90	20.89	***	2/41.56	.14	.870	2/41.98	8.31	***
Group × Gender	1/43.02	3.02	.090	1/43.01	82.37	***	1/43.03	32.58	***
Group × Condition	2/41.96	1.38	.260	2/41.87	9.62	***	2/41.98	3.40	***
Gender × Condition	2/41.11	.97	.390	2/41.17	16.66	***	2/41.75	6.95	.002
Group × Gender × Condition	2/42.07	.34	.720	2/42.07	18.94	***	2/42.03	11.16	***

^*a*^gynephilic subjects vs. androphilic subjects.

^*b*^conventional vs. immersive vs. immersive+.

^*c*^female virtual characters vs. male virtual characters.

^*d*^Kenward-Roger approximation.

*** *p* <.001.

### Sexual Attractiveness of virtual characters

**Fig 3:**
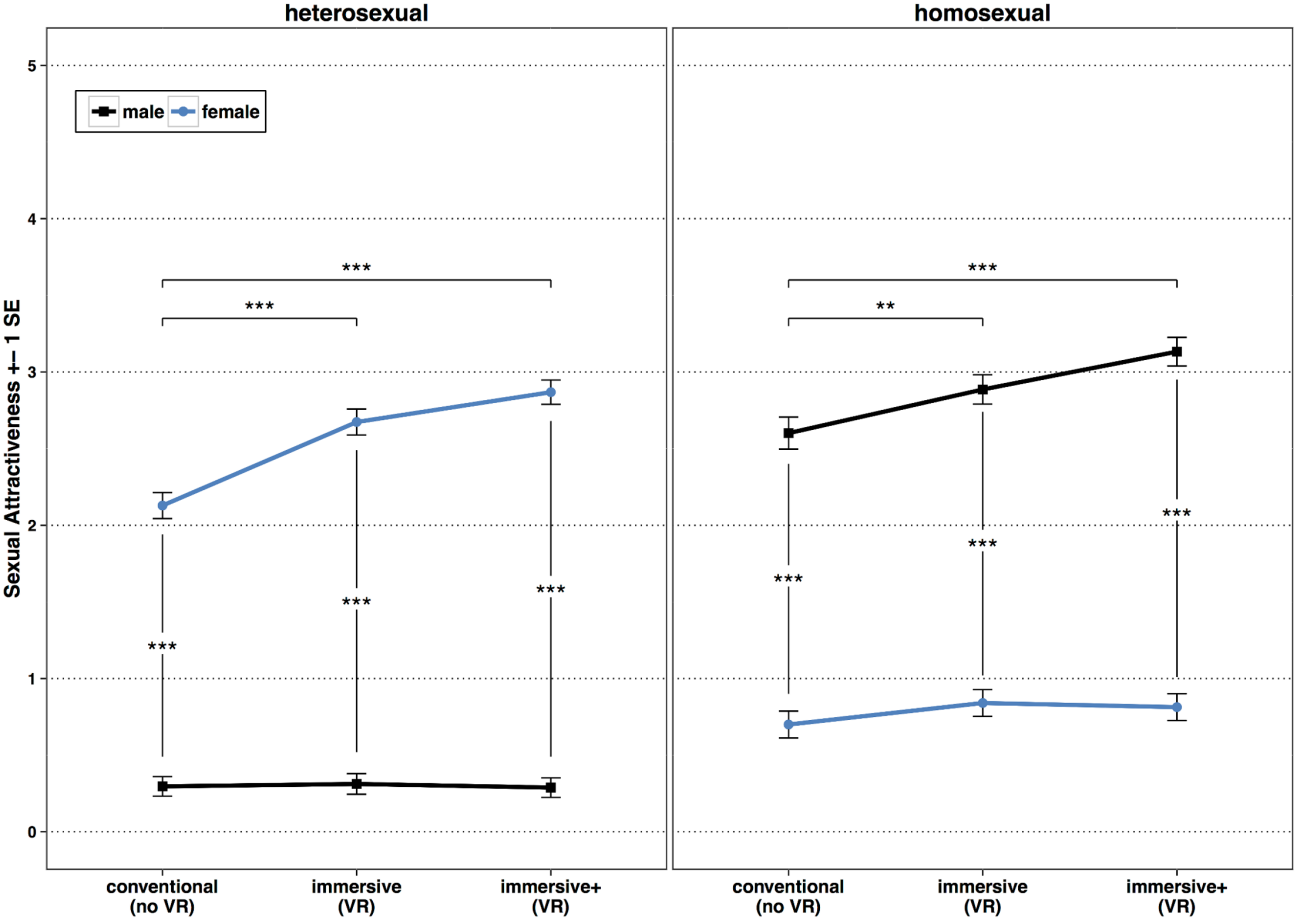
Means and SDs of sexual attractiveness ratings as a function of subject Group, Condition, and Gender of virtual character. Sexual attractiveness rating comprised a 6-point Likert scale ranging from 0 (not sexually attractive) to 5 (very sexually attractive). Blue lines represent mean sexual attractiveness ratings for female virtual characters, black lines mean sexual attractiveness ratings for male virtual characters. * *p* <.05, ** *p* <.01, *** *p* <.001.

Means and SDs for the subjective ratings of the realism of the virtual characters are provided in Table 5, the results of the linear mixed model in Table 6. The linear mixed model revealed a significant three-factorial Group × Gender of virtual character × Condition interaction, *F*(2,42.07) = 18.94, *p* <.001. Fig 3 shows the significant three-factorial Group × Gender × Condition interaction and the results of the post-hoc pairwise comparisons. Post-hoc pairwise comparisons showed for each experimental condition that gynephilic subjects rated female characters as significantly more sexually attractive than male characters, *conventional*: *t*(63.94) = -5.89, *p* <.001, *d* = 1.47, *immersive*: *t*(63.13) = -7.70, *p* <.001, *d* = 1.94, *immersive+*: *t*(62.78) = -7.70, *p* <.001, *d* = 1.94. Androphilic subjects rated male characters as significantly more sexually attractive than female characters in each condition, *conventional*: *t*(62.49) = 5.50, *p* <.001, *d* = 1.39, *immersive*: *t*(61.33) = 5.75, *p* <.001, *d* = 1.47, *immersive+*: *t*(61.12) = 7.45, *p* <.001, *d* = 1.91.

Gynephilic subjects differed in their sexual attractiveness ratings between the experimental conditions only regarding female characters, androphilic subjects ratings differed only regarding male characters. Gynephilic subjects rated female characters in the *immersive+* condition, *t*(45.62) = -7.30, *p* <.001, *d* = 2.16, as well as in the *immersive* condition as more sexually attractive than in the *conventional* condition, *t*(46.24) = -5.41, *p* <.001, *d* = 1.59. However, there was no significant difference between the *immersive+* and the *immersive* condition, *t*(50.39) = -2.47, *p* = .158, *d* = .70. Androphilic subjects rated male characters in the *immersive+* condition, *t*(44.90) = -5.99, *p* <.001, *d* = 1.80, as well as in the *immersive* condition as more sexually attractive than in the *conventional* condition, *t*(43.05) = -3.62, *p* = .008, *d* = 1.10. Nevertheless, there was only a borderline significant trend between the *immersive+* and the *immersive* condition, *t*(43.80) = -2.83, *p* = .072, *d* = .86.

### Viewing Time

**Fig 4:**
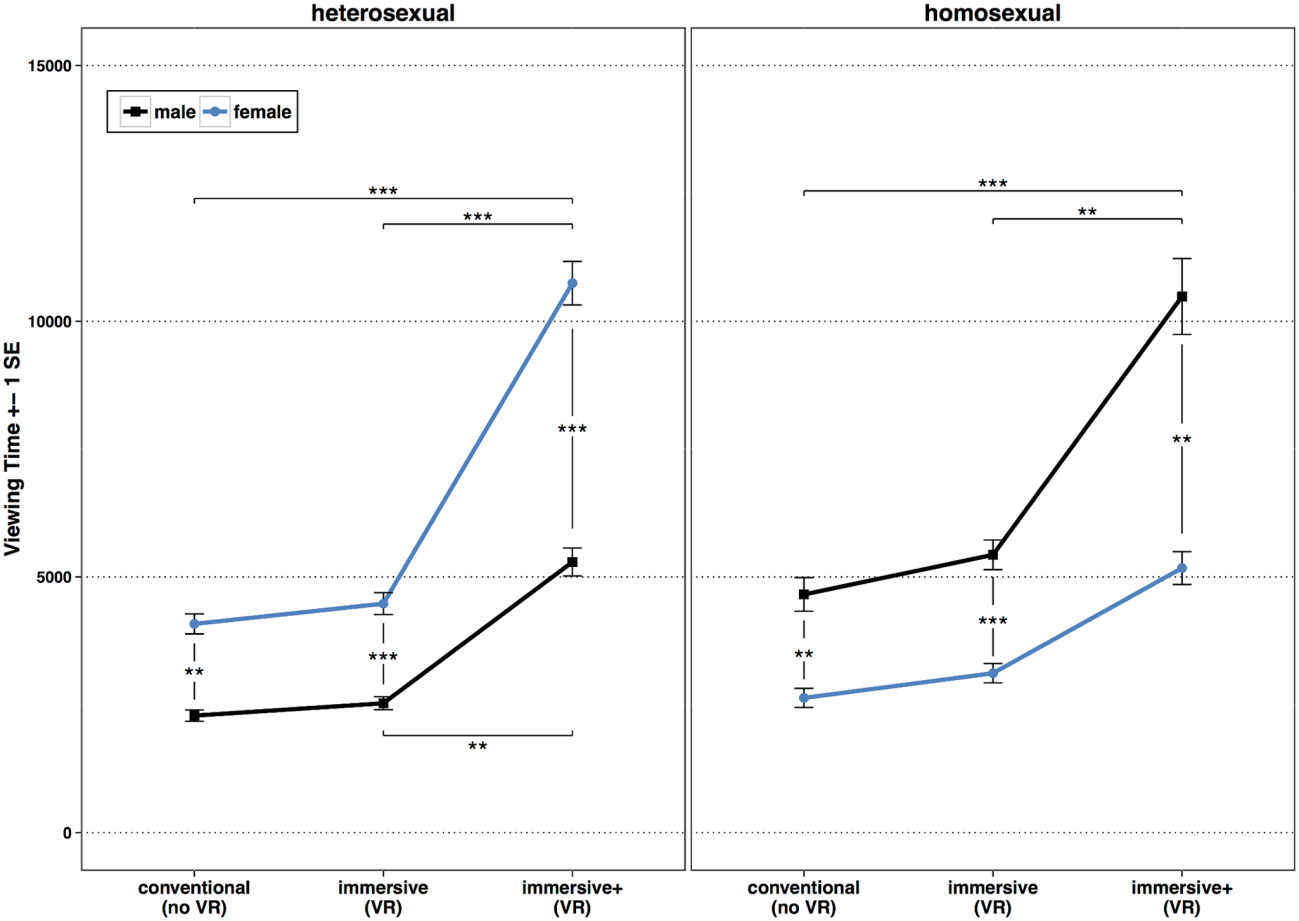
Means and SDs of the viewing time in milliseconds (ms) as a function of subject Group, Condition, and Gender of virtual character. Blue lines represent mean viewing times for female virtual characters, black lines mean viewing times for male virtual characters. * *p* <.05, ** *p* <.01, *** *p* <.001.

Means and SDs for the viewing time are provided in Table 5, all results of the linear mixed model in Table 6. The linear mixed model revealed a significant three-factorial Group × Gender of virtual character × Condition interaction, *F*(2,42.03) = 11.16, *p* <.001. Fig 4 shows the significant three-factorial Group × Gender × Condition interaction and the results of the post-hoc pairwise comparisons. Post-hoc pairwise comparisons showed in line with hypothesis 3, that gynephilic participants looked significantly longer at female than at male characters. This holds true for all three experiments, *conventional*: *t*(48.52) = -3.91, *p* = .002, *d* = 1.12, *immersive*: *t*(50.04) = -4.11, *p* <.001, *d* = 1.16, *immersive+*: *t*(47.79) = -4.95, *p* <.001, *d* = 1.43. Androphilic subjects looked significantly longer at male characters than at female characters in all three experimental conditions, *conventional*: *t*(48.74) = 3.94, *p* = .001, *d* = 1.13, *immersive*: *t*(49.97) = 4.40, *p* <.001, *d* = 1.24, *immersive+*: *t*(47.83) = 3.40, *p* = .002, *d* = .98.

For gynephilic subjects post-hoc pairwise comparisons showed, regarding the viewing time for male characters, a significant difference only between the *conventional* condition and the *immersive+* condition, *t*(46.79) = -3.89, *p* = .004. Viewing times for female characters differed significantly between the *conventional* and the *immersive+* condition, *t*(46.71) = -8.59, *p* <.001, *d* = 2.51, as well as between the *immersive* and the *immersive+* condition, *t*(46.82) = -7.91, *p* <.001, *d* = 2.31. There was no significant difference between the *immersive* and the *conventional* condition, *t*(45.87) = -.93, *p* = .936, *d* = .27. Androphilic subjects showed no significant differences in their viewing times for female characters between the three experimental conditions. However, they looked longer at male characters in the *immersive+* condition than in the *conventional* condition, *t*(46.93) = -6.52, *p* <.001, *d* = 1.90, or in the *immersive* condition, *t*(47.07) = -4.30, *p* = .001, *d* = 1.25. There was no significant difference between the *immersive* and the *conventional* condition, *t*(46.80) = -.86, *p* = .948, *d* = .25.

### Turn arounds in the *immersive+* condition

In order to analyze to what extend subjects turned around the virtual characters in the *immersive+* condition, the sum of degrees subjects turned around each character was analyzed. The linear mixed model revealed a significant Group × Gender interaction, *F*(1,43.08) = 38.62, *p* <.001. Post-hoc pairwise comparisons showed that gynephilic subjects turned significantly more often around female characters (*M* = 344.24, *SD* = 291.29) than around male characters (*M* = 141.74, *SD* = 163.86), *t*(63.82) = -7.10, *p* <.001, *d* = 1.78. Androphilic subjects turned significantly more degrees around male (*M* = 294.05, *SD* = 183.64) than female characters (*M* = 143.22, *SD* = 144.91), *t*(62.22) = -6.01, *p* <.001, *d* = 1.52.

### Sexual orientation index (SOI) and discriminant validity

The Sexual Orientation Index (SOI) was defined as the difference between the viewing time for female characters and the viewing time for male characters. Table 7 shows means and SDs of the sexual orientation index as a function of subject Group and Condition. The linear mixed model revealed a significant Group × Condition interaction, *F*(2,86) = 17.83, *p* <.001. Post-hoc pairwise comparisons revealed that the SOI was, in all three experimental conditions, significantly higher in the gynephilic group than in the androphilic group (*conventional*: *t*(109.02) = 3.17, *p* = .002, *d* = 2.16; *immersive*: *t*(109.02) = 3.67, *p* <.001, *d* = 1.63; *immersive+*: *t*(109.02) = 9.14, *p* <.001), *d* = 1.78. Furthermore, the SOI was, in the gynephilic group, higher in the *immersive+* condition than in the *conventional* condition, *t*(94.19) = 4.26, *p* <.001, *d* = .88, or in the *immersive* condition, *t*(94.19) = 4.01, *p* <.001, *d* = .83. There was no significant difference between the *conventional* and the *immersive* condition, *t*(94.19) = .24, *p* = .969, *d* = .05. Androphilic subjects showed a significantly lower SOI in the *immersive+* condition than in the *conventional* condition, *t*(94.19) = 3.42, *p* = .003, *d* = .70, or in the *immersive* condition, *t*(94.19) = 3.03, *p* = .009, *d* = .62. There was again no difference between the *conventional* and the *immersive* condition, *t*(94.19) = .39, *p* = .920, *d* = .08.

**Table 7:** Means and SDs for the Sexual orientation Index (SOI) as a function of subject Group and experimental Condition.

Condition	gynephilic		androphilic	
	*M*	*SD*	*M*	*SD*
conventional	1744.04	2077.87	-1966.37	1979.63
immersive	1952.73	2197.00	-2344.06	2423.16
immersive+	5426.55	5068.69	-5271.09	7035.39

The SOI is defined as the difference between the viewing time for virtual female characters and the viewing time for virtual male characters. The SOI is shown in milliseconds (ms).

In order to test how well the three experiments can distinguish regarding the sexual orientation of the subjects, ROC analyses were performed based on the SOI. ROC analysis revealed an AUC of.952 (95%-CI:.893—1.00) for the *conventional* condition. Using a cut-off criterion of -100.65 ms, the *conventional* condition differed between gynephilic and androphilic subjects with a sensitivity of 95% and a specificity of 88%. ROC analysis demonstrated an AUC of.944 (95%-CI:.882—1.0) for the *immersive* condition, resulting in a sensitivity of 90% and a specificity of 88% (by using a cut-off criterion of -34.93 ms). Finally, ROC analysis revealed an AUC of.962 (95%-CI:.909—1.00) for the *immersive+* condition. Using a cut-off criterion of -578.11 ms, the *immersive+* condition demonstrated a sensitivity of 90% and a specificity of 96%. From a statistical point of view, the discriminant validity of *conventional* condition did not differ from the discriminant validity of the *immersive* condition, *Z* = -0.19, *p* = .853, or the *immersive+* condition, *Z* = -.273, *p* = .783. Also, there was no significant difference between the *immersive* and the *immersive+* condition, *Z* = .425, *p* = .671.

## Discussion

To our knowledge this was the first study exploring the impact of presence on objective sexual interest and subjective sexual attractiveness using a high-immersive VR system. One aim of the study was to explore the influence of presence on sexual attractiveness of virtual male and female characters. According to our hypothesis, stereoscopic viewing and the possibility to interact with the environment should result in a higher sense of presence. The second aim of the study was to test wether higher realism and higher sexual attractiveness of virtual characters can enhance the viewing time effect. In order to do so, we compared a conventional viewing time experiment (non-stereoscopic viewing, no interactivity) with two different experiments in which the viewing time method was used in combination with virtual reality: in the *immersive* condition, the virtual characters were presented with an HMD allowing stereoscopic viewing and head tracking. In the *immersive+* condition, the participant additionally had the possibility to turn around virtual characters.

### Presence and Simulator Sickness

First of all, neither wearing a HMD nor turning around a virtual human character within the VE resulted in more pronounced simulator sickness symptoms. Therefore, the study shows that viewing virtual human characters in high-immersive VE for the *immersive+* does not pose, from a medical point of view, more risk for the participant than viewing human characters on a standard desktop system. This result is a little surprising since a large amount of previous studies mentioned simulator sickness as one of the major disadvantages of HMDs [32, 71]. One possible explanation could be that the participants in our study did not have to move over wide distances within the VE as it was necessary in most other studies demonstrating the negative effect of HMDs on simulator sickness.

The experimental manipulation of immersion resulted in significantly higher values of Spatial Presence, Realness, Involvement and the general item of the IPQ in both subject groups: Stereoscopic Viewing lead to a higher overall sense of presence. However, contrary to hypothesis 1, the feeling of being there did not further increase with the additional possibility to interact with the virtual environment. The enhancement of the sense of presence due to stereoscopy is in line with the already mentioned literature regarding presence and the concept of presence [22, 24, 25, 27, 72]. The surplus on interactivity significantly influenced only the sub scale Involvement of the IPQ. Gynephilic and androphilic subjects experienced the highest Involvement in the *immersive+* condition. Involvement measures, how focused the subject is on the VE and how much he suppresses the real environment [17]. Thus, suppression of the real world seems to work better with stereoscopic viewing, but best when the subject has the additional possibility to interact with the virtual environment. The sub-scale Realness measures the realness of the virtual environment in comparison to the real world [17]. Thus, only stereoscopic viewing, but not the possibility to interact with the virtual environment, enhanced the Realness of the virtual environment over the entire course of the experimental conditions. Nevertheless, it is important to note, that the IPQ sub-scale Realness is related to the entire virtual environment, not only to the virtual human characters. Unfortunately, the IPQ does not allow differentiating between different aspects (e.g. human characters, stage) of the virtual environment regarding Realness.

### Subjective realism of virtual characters

The realism of the virtual characters was additionally assessed with self reports. In comparison to the IPQ sub scale Realness, subjective ratings of realism of virtual characters showed that stereoscopic viewing as well as interactivity enhanced the realism of virtual characters. Since realism involves the comparison between the virtual environment and the real world [11, 17, 18], a higher subjective realism of sexual stimuli can be interpreted as a higher ecological validity of the two high-immersive conditions in comparison to viewing virtual sexual stimuli on a standard desktop PC. Realism of virtual characters can be seen as another dimension of presence, social presence or co-presence. Social presence “reflects how users immersed in VR feel that virtual humans are really there, in the room, with them.” (p. 62) [73]. Social presence can be divided into two concepts, one focusing on the perception of embodied virtual characters and one on the social responses to the virtual characters [74]. The subjective realism of virtual characters refers to the first concept, since it asks rather about the perception of the virtual character than about social interactions. Social presence seems to be mediated not only by the amount of immersion or anthropomorphism (realism of shape and textures of the character) but also by the degree of behavioral realism, the interaction between anthropomorphism and behavioral realism, the belief of the subject about the nature of the virtual character, and observer characteristics [75]. Some studies reported that more realistic looking virtual characters (in a sense of more human-like anthropomorphism) can enhance social presence [76]. Yet, other studies showed the so called “uncanny valley” effect: the more realistic a human virtual character, the lower the tolerance for (even small) imperfections in behavioral realism. It could be that a highly realistic virtual character results in a lower social presence than a minor realistic character due to these small imperfections [73–75]. Nevertheless, it is important to note that realism of virtual characters is only one factor influencing social presence and we did not assess social presence or co-presence in a direct manner. The realism of the virtual characters was not in the main focus of our study either and we did not manipulate the realism of the characters. All characters were identical in all three experimental conditions. Thus, our study design does not allow reasoning about social presence, as it is defined above, or about the impact of realism on social presence. Furthermore, subjective rating of realism always took place before the assessment of general presence. Thus, the subjective realism rating could have influenced the presence rating.

### The influence of presence on sexual attractiveness of virtual characters

Stereoscopic viewing and interactivity comes along with a significantly higher subjective sexual attractiveness of sexual relevant virtual human characters: Gynephilic participants rated the same female characters as significantly more sexually attractive when they saw them in the *immersive* and the *immersive+* condition, compared to the *conventional* condition. The same holds true for androphilic participants and male virtual characters. Since the presence ratings were also higher in these experimental conditions, one could hypothesize that a higher presence results in a higher subjective sexual attractiveness of virtual characters. This is, to our knowledge, the first direct empirical evidence that stereoscopic viewing and interactivity enhance the subjective sexual attractiveness of non-animated virtual human characters. Furthermore, it seems to be the first direct empirical verification for the predominance of high-immersive virtual environments over conventional two-dimensional stimuli in the context of sexuality research. Still, the additional possibility to interact with the virtual characters in the *immersive+* condition did not enhance the sexual attractiveness of sexually relevant characters. Subjects reported only a higher Involvement in the *immersive+* condition, but no higher overall sense of presence or higher realism of the virtual characters. Thus, one can hypothesize that a higher involvement or interactivity does not result in a higher sexual attractiveness of virtual characters. Its is important to note, that the possibility to turn around virtual characters involves the subject not as much as, for example, the possibility to talk to virtual characters as it will be possible in more sophisticated virtual environments.

Indirect evidence for a higher sexual attractiveness due to a higher sense of presence already exists. Renaud et al. [37], for example, compared sexual arousal, measured by PPG, caused by computer-generated human characters in an immersive virtual environment and by auditory sexual scripts. 42 healthy gynephilic and androphilic participants as well as 22 child abusers took part in the study. AUC analyses based on PPG measures pointed out, that the classification of child abusers and non child abusers was significantly better when using virtual characters compared to auditive scripts. Thus, immersive virtual environments in combination with PPG seem to outplay conventional PPG measurement methods. However, the study lacks a direct comparison of visual two-dimensional stimuli with three-dimensional stimuli in an immersive virtual reality. The predominance of high-immersive virtual environments over two-dimensional non-immersive environments regarding their ability to induce emotions has been in the direct focus of emotion research and is well documented. Since sexual arousal as well as other sexual experiences can be defined as an emotion, these studies directly support the results of our study [35]. For example, a study of Kim et al. [32], intensively described in the introduction, showed, that VR systems enabling stereoscopic viewing induce significantly higher emotional arousal than VR systems without stereoscopic viewing (e.g. desktop systems). Also Gorini et al. [5] pointed out the predominance of VR over 2D desktop systems regarding emotion induction based on a sample of 20 patients with eating disorders and healthy subjects. The authors compared anxiety responses on real food, virtual food viewed in a high-immersive VE, and pictures of food. Real food and virtual food elicited comparably high anxiety responses which were significantly higher than those found for 2D food viewed on a standard desktop system. The authors concluded that the higher the sense of presence the higher the emotional response. Further evidence for the predominance of 3D stimuli over 2D stimuli comes from neurobiological emotion research. For example, Dores et al. [77] compared the brain activation elicited by two- and three-dimensional emotional stimuli. The results showed increased brain activation for the 3D affective-inducing stimuli in comparison with the same stimuli in 2D scenarios, mostly in cortical and subcortical regions that are related to emotional processing. Thus, one can assume that 3D stimuli increase induced emotions. On the other hand, the enhancement of emotion induction due to stereoscopic viewing is discussed controversially in the literature. For example, Banos et al. [26] immersed 40 healthy participants in two experimental (stereoscopy vs. monoscopy) and two emotional conditions (relaxation vs. joy) within a virtual environment. The virtual environment consisted of a park, the emotional conditions were achieved with different sounds and visual elements (shadows, lights, textures). Stereoscopic as well as non-stereoscopic presentation of the virtual environment induced positive moods. However, there were no significant differences between stereoscopic and non-stereoscopic viewing regarding the experienced amount of either relaxation or joy. The results denote the lack of impact of stereoscopy on the intensity of emotional reactions elicited in virtual environments. Regarding negative emotions, comparable results are reported. Morina et al. [78] explored the impact of stereoscopic viewing on the sense of being there and the anxiety within social interaction in a non-clinical sample of 38 participants. The participants were randomly assigned to either an HMD enabling stereoscopic viewing or a one-screen projection based system without stereoscopic viewing. The virtual environment involved social interactions with a virtual human controlled by research assistants. Participants reported significantly higher levels of presence in the stereoscopic condition than in the non-stereoscopic condition. Nevertheless, there was no difference regarding self reported anxiety between the two conditions. Therefore, stereoscopy seems to have no influence on the degree of experienced anxiety in virtual environments. Since the stereoscopic condition also introduced a higher amount of interactivity due to head tracking, it is possible that interactivity, and not stereoscopy, is the most important factor. Unfortunately, the study does not allow to differentiate between the impacts of stereoscopy and interactivity in the stereoscopic condition. Still, its is important to bear in mind, that the causal relationship between presence and induced emotions still remains unclear and neither our study cannot solve this causal relationship.

The subjective rating data are a first evaluation of the 3D stimulus set which was used in our study. To our knowledge, there only exist two other sets of three-dimensional human figures used in sex research. Renaud et al. [79] developed a set of 3D virtual characters depicting realistic naked human individuals for clinical forensic rehabilitation of sex offenders. To our knowledge, the set consists of only one girl, one boy, one woman, and one man. The stimulus set is well validated regarding the age of the virtual characters and its ability to induce sexual interest. From an experimental point of view, the small number of stimuli is a major limitation of this stimulus set. Another stimulus set, the Virtual People Set (VPS), consists of 108 computer-generated human figures varying in terms of gender, explicitness, and physical maturity [80]. The human figures were modeled three-dimensionaly, but the VPS only consists of rendered and color corrected 2D images of these models. The 2D stimuli are well validated, but 2D stimuli do not match the needs of high-immersive VR applications. Therefore, our set of 10 female and 10 human characters seems to be, from an experimental point of view, a fundamental enhancement of existing three-dimensional stimulus sets in the context of sex research.

### The impact of presence on viewing time

In line with hypothesis (3), androphilic participants looked significantly longer at male than at female virtual characters. Gynephilic subjects, on the other hand, looked significantly longer at female than male virtual characters. This viewing time effect was existent in all three experimental conditions. Thus, there seems to be evidence that the viewing time paradigm can be successfully transferred into high-immersive VR applications. It further emphasizes the usability of the developed virtual human characters for sex research: Since subjects did not know that the viewing time was assessed, the tendency of participants to answer in a socially desirable manner may not have influenced the viewing time results. Thus, viewing time is a covert measure of sexual interests and validates subjective ratings of sexual attractiveness [48]. Therefore, one may assume that the subjective rating data is valid and not influenced by social desirability. Since socially desirable behavior was not assessed or controlled in our study, this assumption has to be tested in a direct manner in future experiments.

Contrary to hypothesis (4), a higher sense of presence or a higher sexual attractiveness of the virtual characters does not seem necessarily to result in a higher Sexual Orientation Index (SOI). On the contrary, the SOI was significantly enhanced only by the additional possibility to turn around the virtual characters, not by stereoscopic viewing and fundamental interactivity provided by head tracking. At first sight, a longer viewing time in the *immersive+* condition may be easily explained: Turning around virtual characters consumes time. Thus, longer viewing times in the *immersive+* condition are probably artifacts of the possibility to interact with the VE. If the use of turning around the character is unrelated to the perceived sexual attractiveness of the virtual characters, subjects should have turned around female characters just as often as they did with male characters. Yet, an analysis of the sum of degrees subjects turned around virtual characters showed that gynephilic subjects turned significantly more degrees when they were presented with female characters than they did with male characters. In contrast, androphilic subjects turned significantly more degrees around male than female characters. Thus, one can assume that the use of interactivity depends on the sexual attractiveness of the virtual character: sexually attractive characters are observed more intensively than sexually non attractive characters. Thus, the possibility to interact with the virtual environment seems to result in a more pronounced viewing time effect and may be more than only an artifact.

In order to explain the lacking significant difference between the *conventional* and the *immersive* condition regarding the SOI, one can assume that task-based effects are important for the viewing time effect rather than stimulus-specific mechanisms. Imhoff et al. [51] argued that the viewing time effect is not based on the fact that people want to look longer at potentially rewarding sexually attractive pictures, but on task-specific mechanism. The authors came to this conclusion since the viewing time effect remains stable, even when the presentation time of the pictures is not under the control of the subject [51]. Furthermore, in their experiments, the authors presented sexually attractive and sexually non attractive pictures for 750 and 500 ms, respectively, and the attractiveness rating was performed after the stimulus presentation. By separating the rating from viewing the pictures, it was possible to explore the influence of the stimulus on viewing time, separated from the influence of the task. Since the viewing time effect also existed when the rating was performed in the absence of the stimulus, the hypothesis, that it results from automatic attentional processes which are elicited by the stimulus, seems not preservable. Thus, the authors assume two possible explanations for the viewing time effect: (1) time-consuming schematic processes triggered by sexually attractive stimuli, which distract participants from the rating task and (2) cognitive demands associated with the task of rating sexual attractiveness (e.g. deciding on the physical features or the sex of the target person) [52]. In a recent study, 32 gynephilic and 32 androphilic men had the task to take different perspectives while performing the viewing time task [52]. The different perspectives comprised the rating of the stimuli from the point of view of a gynephilic man/woman and a androphilic man/woman. The rationale behind this experiment was that if only the stimuli triggered the viewing time effect, then the task should be irrelevant. Results showed a task-based effect as well as a stimulus-based effect. Based on effect sizes, task-specific processes seemed to be more pronounced than stimulus-specific mechanisms. The results of our study, too, seem to support the hypothesis of a greater influence of task-specific effects on the viewing time than stimulus-driven effects. If stimulus-driven effects would be most influential, then a higher subjective sexual attractiveness should result in longer viewing times [52].

From a more clinical point of view, using high-immersive VR systems makes it possible to distinguish androphilic and gynephilic men just as much as conventional viewing time measures on standard desktop systems. The ROC analyses provided sensitivity and specificity values for all three experimental conditions comparable to or better than previous results. For example, Israel et al. [53] explored the viewing time as an objective measure of sexual interest in gynephilic men and women. Based on a sample of 51 gynephilic men and 55 gynephilic women, the authors identified a sensitivity of 90% based on discriminant analysis. Only 3.6% of all women were misclassified as men, whereas 15.7% of all men were falsely classified as women. In another study, 52 androphilic men and 47 gynephilic women took part in a viewing time experiment. Classification analyses revealed a sensitivity of 88% [54]. Even though the *immersive+* condition provided the highest classification accuracy, classification accuracy did not differ significantly between the three conditions, from a statistical point of view. This is contrary to hypothesis (5). Considering the already almost perfect AUC value in the *conventional* condition, the lack of a statistically significant difference is not surprising: It is much more difficult to improve the classification accuracy at a very high baseline level than at a low baseline level. Nevertheless, the viewing time method seems to be unable to benefit from the most important advantages of VR: The possibility to induce higher sexual interest than conventional two-dimensional pictures and a higher ecological validity due to a higher realism of stimuli. Other already existing paradigms can possibly benefit more from VR technology. For example, the eye-tracking method [45, 81, 82] seems to be a potential candidate for VR systems since its effects (eye movements) are primarily based on the used stimuli. However, further studies are necessary to explore the possible benefits of VR systems in the clinical context of sex research.

### Limitations

Several limitations have to be mentioned. First, the used HMD suffers from a so called “screen door mesh effect” resulting in visible black lines between the single pixels of the screen. This effect occurred since the HMD uses lenses which magnify the screen implemented in the HMD. This screen door mesh effect was not visible on the conventional desktop system. Thus, despite the same resolution of both screens, the sharpness of the conventional desktop was higher than the sharpness of the HMD screen. This fact could have significantly influenced the results of the study. It must be assumed, that a sharper screen would result in a higher realism of presented stimuli. Perhaps, the difference between the desktop system and the VR system regarding realism of characters was lowered due to the screen door mesh effect. Nevertheless, the results showed that realism was lowest on the desktop system. This difference would probably be more pronounced without the “screen door mesh effect”. Second, only stereoscopy and interactivity were experimentally manipulated as factors which could possibly have an influence on presence. However, it is well known that other factors, besides technological aspects, can also influence the sense of presence, especially factors based in the experiences and personality of the individual subject. For example, previous experiences with computer games and attitudes towards computers can enhance or decrease the subjective sense of presence [11]. Another factor, which could get important in this respect, is the fact, that our sample consisted of young men who grew up with high sophisticated video games. This fact probably could have heightened the importance of realism of the virtual characters especially given the provided possibilities to interact with the virtual environment. Since these factors were not assessed, a possible influence of such variables on the reported results cannot be eliminated. Third, the sense of presence was assessed with a self-report questionnaire. Studies pointed out that for example prior experience in rating stimuli on other aspects such as interest and 3D-ness can significantly influence the subsequent presence rating. Furthermore, different presence questionnaires can result in different presence ratings due to different definitions of presence used in the questionnaires [11]. Thus, results of this study regarding presence have to be limited to the mentioned presence concept of [22]. Fourth, although the study immersed the subjects with a virtual character, social presence was not assessed. Since social presence could be an important dimension of presence in our study, further research should assess social presence in a direct manner in order to explore this dimension of presence in more detail. Fifth, we only assessed the sexual behavior of the subjects but not the sexual attraction. In further studies, sexual attraction should be used to characterize the sexual orientation of men.

## Conclusions

In summary, the study showed the potentials of high-immersive VR for sex research. Moreover, it shows that VR can enhance the realism and subjective sexual attractiveness of sexually relevant stimuli. Furthermore, measures of objective sexual interest in high-immersive VE are as valid as they areon conventional desktop systems. Therefore, one can assume that VR is able to enhance the ecological validity of experiments and measures in the context of sex research. From a clinical point of view, the current experimental design is not able to enhance the discriminant validity of the viewing time method in a significant manner. In this study, a very simple VE was used. More sophisticated VE can probably enhance the reported effects. For example, our study used only static virtual characters. An interesting question would be, if animating the characters could further enhance the ecological validity. For example, Regenbrecht et al. [22] already pointed out that animations can significantly enhance the realism of the the VE. Since the hardware for VR applications is getting cheaper and cheaper, and facing the obvious advantages of VR in the study of human behavior (e.g. high ecological validity), VR applications provide new possibilities for sex research.
